# HRV Features as Viable Physiological Markers for Stress Detection Using Wearable Devices

**DOI:** 10.3390/s21082873

**Published:** 2021-04-19

**Authors:** Kayisan M. Dalmeida, Giovanni L. Masala

**Affiliations:** Department of Computing and Mathematics, Manchester Metropolitan University, Manchester M15 6BH, UK; kayisan.m.dalmeida@stu.mmu.ac.uk

**Keywords:** stress, wearable device, machine learning, smart watch, heart rate variability, electrocardiogram

## Abstract

Stress has been identified as one of the major causes of automobile crashes which then lead to high rates of fatalities and injuries each year. Stress can be measured via physiological measurements and in this study the focus will be based on the features that can be extracted by common wearable devices. Hence, the study will be mainly focusing on heart rate variability (HRV). This study is aimed at investigating the role of HRV-derived features as stress markers. This is achieved by developing a good predictive model that can accurately classify stress levels from ECG-derived HRV features, obtained from automobile drivers, by testing different machine learning methodologies such as K-Nearest Neighbor (KNN), Support Vector Machines (SVM), Multilayer Perceptron (MLP), Random Forest (RF) and Gradient Boosting (GB). Moreover, the models obtained with highest predictive power will be used as reference for the development of a machine learning model that would be used to classify stress from HRV features derived from heart rate measurements obtained from wearable devices. We demonstrate that HRV features constitute good markers for stress detection as the best machine learning model developed achieved a Recall of 80%. Furthermore, this study indicates that HRV metrics such as the Average of normal-to-normal (NN) intervals (AVNN), Standard deviation of the average NN intervals (SDNN) and the Root mean square differences of successive NN intervals (RMSSD) were important features for stress detection. The proposed method can be also used on all applications in which is important to monitor the stress levels in a non-invasive manner, e.g., in physical rehabilitation, anxiety relief or mental wellbeing.

## 1. Introduction

Stress can be defined as a biological and psychological response to a combination of external or internal stressors [1,2], which could be a chemical or biological agent or an environmental stimulus that causes stress to an organism [3]. Stress is, in essential, the body’s coping mechanism to any kind of foreign demand or threat. At the molecular level, in a stressful situation the Sympathetic Nervous System (SNS) produces stress hormones, such as cortisol, which then, via a cascade of events, lead to the increase of available sources of energy [4]. This large amount of energy is used to fuel a series of physiological mechanisms such as: increasing the metabolic rate, increasing heart rate and causing the dilation of blood vessels in the heart and other muscles [5], while decreasing non-essential tasks such as immune system and digestion. Once stressors no longer impose a threat to the body, the brain fires up the Parasympathetic Nervous System (PSN) which is in charge of restoring the body to homeostasis. However, if the PSN fails to achieve homeostasis, this could lead to chronic stress; thus, causing a continual and prolonged activation of the stress response [6]. Conversely, during acute stress, the stress response develops immediately, and it is short-lived.

Studies carried out in this field suggest that stress can lead to abnormalities in the cardiac rhythm, and this could lead to arrythmia [7]. Additionally, stress does not only have physical implications, but it can also be detrimental to one’s mental health; in fact, chronic stress can enhance the chances of developing depression. For these reasons, it is important to develop a system that can detect and measure stress in an individual in a non-invasive manner in such way that stress can be regulated or relieved via personalised medical interventions or even by just alerting the user of their stressful state.

Furthermore, stress has been identified as one of the major causes of automobile crashes which then lead to high rates of fatalities and injuries each year [8]. As reported by Virginia Tech Transportation Institute (VTTI) and the National Highway Traffic Safety Administration (NHTSA), lack of attention and stress were the leading cause of traffic accidents in the US, with a rate of ~80%. Therefore, being able to accurately monitor stress in drivers could significantly reduce the amount of road traffic accidents and consequently increase public road safety.

Given that stress is regulated by the Autonomous Nervous System, it can be measured via physiological measurements such as Electrocardiogram (ECG), Galvanic Skin Response (GSR), electromyogram (EMG), heart rate variability (HRV), heart rate (HR), blood pressure, breathe frequency, Respiration Rate and Temperature [9]. These are considered to be an accurate methodology for bio signal recording as they cannot be masked or conditioned by human voluntary actions. However, this study will be mainly focusing on HRV, which is controlled by PSN and SNS; therefore, an imbalance in any functions regulated by these two nervous system branches will affect HRV [10]. HRV is the variation in interval between successive normal RR (or NN) intervals [11]; it is derived from an ECG reading and it is measured by calculating the time interval between two consecutive peaks of the heartbeats [12]. As explained in [11] the RR intervals are obtained by calculating the difference between two R waves in the QRS complex.

HRV can be subdivided into time domain and frequency domain metrics as described in Table 1.

HRV is traditionally obtained from ECG and requires the use of computational software for calculation; this is a process is limited to laboratory or clinical settings and requires a certain degree of technical knowledge for interpretation and calculation. Thanks to the advancement of technology, however, commercially available portable devices and wearables have the capacity to monitor and record HRV measurements. Dobs et al. (2019) performed a systemic review and meta-analysis on the numerous studies that compared the quality of HRV measurements acquired from ECG and obtained from portable devices, such as Elite HRV, Polar H7 and Motorola Droid [13]. Twenty-three studies revealed that HRV measurements obtained from portable devices resulted in a small amount of absolute error when compared to ECG; however, this error is acceptable, as this method of acquiring HRV is more practical and cost-effective, as no laboratory or clinical apparatus are required [13].

Furthermore, the Apple Watch is one of the most best-selling and popular smartwatches in the market. Studies, carried out by Shcherbina and colleagues [14], demonstrated that the Apple Watch was the best HR estimating smartwatch with one-minute granularity and with the lowest overall median error (below 3%) while Samsung Gear S2 reported the highest error. In addition, it is also important to validate the HRV estimation of the Apple Watch. Currently, the best way to obtain RR raw values from the Apple Watch is via the Breathe app developed by Apple. Authors in [15] conducted an investigation that validated the Apple Watch in relation to HRV measurements derived during mental stress in 20 healthy subjects. In this study, the RR interval series provided by the Apple watch was validated using the RR interval obtained from Polar H7 [15]. Successively, the HRV parameters were compared and their ability to identify the Autonomous Nervous System (ANS) response to mild mental stress was analysed [15]. The results revealed that the Apple Watch HRV measurements had good reliability and the HRV parameters were able to indicate changes caused by mild mental stress as it presented a significant decrease in HF power and RMSSD in stress condition compared to the relax state [15]. Therefore, this study suggests that the Apple Watch presents a potential non-invasive and reliable tool for stress monitoring and detection. In this study, raw RR intervals, from beat-to-beat measurements obtained from the Breathe app, are considered for stress classification.

This study is aimed at developing a good predictive model that can accurately classify stress levels from ECG-derived HRV features, obtained from automobile drivers, testing different machine learning methodologies such as K-Nearest Neighbour (KNN), Support Vector Machines (SVM), Multilayer Perceptron (MLP), Random Forest (RF) and Gradient Boosting (GB). Moreover, the models obtained with highest predictive power will be used as a reference for the development of a machine learning model that would be used to classify stress from HRV features derived from heart rate measurements obtained from wearable devices in a unsupervised system-based web application.

The paper is organised as follows. Section 2 provides a discussion of related work conducted in the literature. Section 3 describes the experimental methodology of the study, including a description of the dataset, pre-processing, hyperparameter tuning and the design protocol used for the development of a simple stress detection web application based on Apple Watch derived data. Section 4 presents the experimental results and Section 5 an intensive discussion of the results obtained. Lastly, Section 6 provides the concluding remarks of the study, as well as proposed future work.

## 2. Related Work

As stress level changes so does the HRV and it has been proven that HRV decreases as stress increases [11]. This is possible because HRV provides a measure to monitor the activity of the ANS and, therefore, can provide a measure of stress [16]. Authors in [16] explored the interaction between HRV and mental stress. Here they took ECG recordings during rest and mental task conditions, which was meant to reflect a stressful state. Linear HRV measures were then analysed in order to provide information on how the heart responds to a stressful task. The results demonstrated that the mean RR interval was significantly lower during a mental task than in the rest condition [16]. This difference was significant only when time domain parameters (pNN50) and the mean RR interval were analysed; while the frequency domain measure did not show a significant difference, although there was an elevated LF/HF in the stressed condition [16]. As LF is associated with the SNS and HF with PNS, the increased LF/HF ratio does suggest that there is a higher sympathetic activity in the stress condition compared to the resting state [16].

Furthermore, investigations have been carried out in order to accurately classify stress in drivers via HRV measurements. For example, authors in [17] aimed to classify ECG data using extracted parameters into highly stressed and normal physiological states of drivers. In this study, they extracted time domain, frequency domain and nonlinear domain parameters from HRV obtained by extracting RR intervals from QRS complexes. These extracted features were fed into the following machine learning classifiers: K Nearest Neighbor (KNN), radial basis function (RBF) and Support Vector Machine (SVM. The results showed that SVM with RBF kernel gave the highest results, with 83.33% accuracy, when applied to time and non-linear parameters, while giving an accuracy of 66.66% with frequency parameter [17]. This was in concordance with the result obtained by [16] as the frequency domain parameters did not give a significant difference between rest and mental tasks.

In this study, instead of analysing how each HRV measure is affected by the onset of stress, we took into consideration the combination of both time and frequency domain HRV features and how these aid stress classification with the use of machine learning models. The performance of the machine learning models was evaluated, taking into consideration the following metrics: Area Under Receiver Operator Characteristic Curve (AUROC), Recall/Sensitivity and F1 score, without relying only on accuracy. Furthermore, we detected stress in a non-invasive manner using the Apple Watch, from which we extracted heart rate data, obtained from volunteers subjected to different mental state conditions.

## 3. Materials and Methods

### 3.1. Datasets

The first part of this study consists in the development of a good stress predictive model from ECG-derived HRV measurements. The dataset used was collected at Massachusetts Institute of Technology (MIT) by Healey and Picard [18], which is freely available from PhysioNet [19]. The dataset consists of a collection of multi-parameter recordings obtained from 27 young and healthy individuals while they were driving on a designated route in the city and highways around Boston, Massachusetts. The driving protocol involved a route that was planned to put the driver though different levels of stress; specifically, the drive consisted of periods of rest, highway driving and city driving which were presumed to induce low, medium and high stress, respectively [18]. This investigation measured four types of physiological signals: ECG, EMG, GSR and respiration. The dataset is available in the PhysioNet waveform format containing 18 *.dat* and 18 *.hea* files with a *.txt* metadata file. Each bio-signal *.dat* file contains the original recording for ECG, EMG, GSR, HR and Respiration. As the aim of this study is to classify stress based on HRV metrics, a beat annotation file was created from *.dat* files by using the WQRS tool that works by locating QRS complexes in the ECG signal using and gives an annotation file as the output [20]. The annotation file serves the purpose of extracting RR intervals together with its corresponding timestamp using the PhysioNet HRV toolkit. HRV features were extracted from the RR intervals by splitting the dataset in windows of 30 s. Time domain features were calculated using a C implementation that connects Python to the PhysioNet HRV toolkit and by calling the *get_hrv* method which returns the HRV metrics. While frequency domain metrics were obtained by applying the Lomb Periodogram which determines the power spectrum at any given frequency [21]. GSR signals were used to determine and label the stress states in drivers, as the marker in the dataset was mainly made of missing values. The median GSR values were used as the cut-off point, thus, values above the median were labelled as stress while the values below the median were labelled as no stress. For clarity reasons, this dataset will be referred to as ‘original-dataset’.

The second portion of this investigation aimed to develop machine learning models that would classify stress from HRV features derived from HRV measurements obtained from the Apple Watch. For this purpose, data was collected from 4 Apple Watch users, who were asked not to exercise or intake caffeine before and on the day of the experiment. The volunteers were subjected to 2 different conditions. The first condition was a 15-min relaxation period where they listened to relaxing lo-fi music. The second was a stressful condition experienced after an 8-h shift of work. Immediately after each task, the volunteers were asked to record their beat-to-beat measurements 5 times, using the Apple Breathe App available on their Apple Watch. The subjects were subjected to these two conditions on separate days. Thanks to the Breathe App, it was possible to obtain raw RR intervals from beat-to-beat measurements and all the data was accessible from the user’s Personal Health Record, which can be exported in XML format via Apple’s Health App. The beat-to-beat measurements of interests, mapped into the <InstantaneousBeatsPerMinute> tag, were extracted from the XML file in Python using *xml* and *pandas* modules. Successively, the raw RR intervals (in seconds) were derived from the beat per minute (bpm) readings using the following equation:

(1) $$RR=\frac{60}{bpm}$$

**Figure 1:**
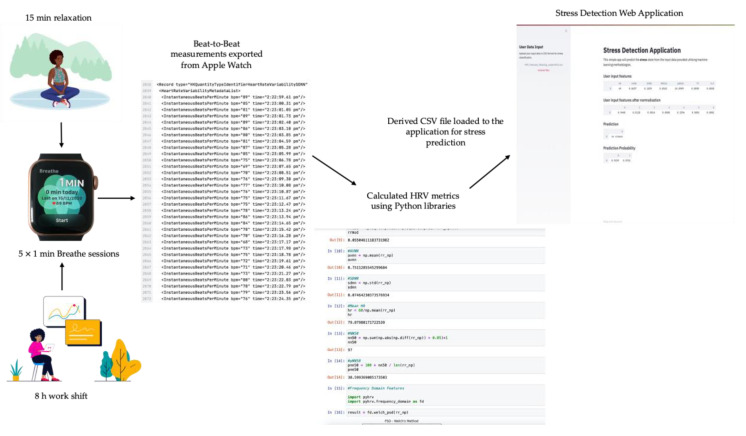
Illustration of the experimental procedure followed for stress detection on data obtained from Apple Watch users.

Moreover, HRV features were extracted from the calculated *RR* intervals using the *NumPy* library, for time domain, and the *pyhrv* library, specifically the *frequency_domain* module and the Welch’s Method for frequency domain features [22]. This dataset will be used as a blind test for the obtained classifier, in order to measure its predictive power on unseen data; hence, this dataset will be referred to as a ‘blind-dataset’ throughout this paper. The stress prediction of the blind-dataset was performed by a simple web application, developed using *Streamlit*. This experimental procedure is illustrated in Figure 1.

#### 3.1.1. Data Pre-Processing

Firstly, missing values in original-dataset were replaced with the mean value of each column. Then the data was further split into training and testing datasets with an 80:20 (training:testing) split. From this point onwards, the testing and training data were treated separately as different entities in order to prevent data overfitting and data leakage. Data normalisation was done separately on the training and testing set instead of the whole dataset that could leak information about the test into the train set. Normalisation was performed using the *scikit-learn* library, where continuous values are rescaled in a range between 0 and 1 with the aim of having all numeric columns in the same range, as there are features that are in different ranges such as ECG, HR, EMG, seconds and HF.

#### 3.1.2. Feature Selection

**Figure 2:**
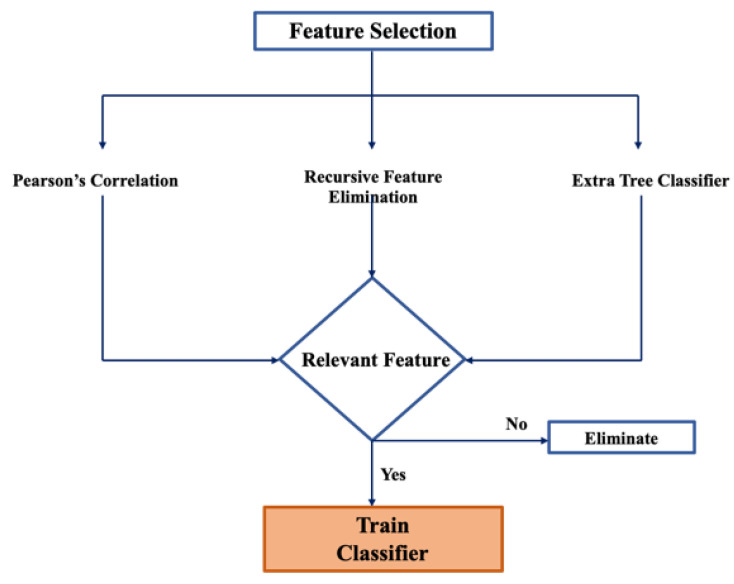
Flow chart illustrating the Feature Selection Process implemented in this study.

Features were selected based on their relevance to the classification task that this study proposed. This was accomplished using three techniques: Pearson’s Correlation, Recursive Feature Elimination (RFE) [23] and Extra Tree Classifier [24], used to estimate feature importance. The common least important features from each method were dropped from both training and testing datasets; Figure 2 illustrates this process.

Pearson’s Correlation calculates the correlation coefficient between each feature and the target class (stress) and this value ranges between −1 and 1. Low correlation is represented by values close to 0, with 0 being no correlation, and high positive and negative correlations are achieved with values closer to 1 and −1, respectively. In this study, relevant features were chosen based on their highly positive and highly negative correlations with the target. Feature Importance using Extra Trees Classifier, is an ensemble-based learning algorithm that aggregates the results of multiple decision trees to output a classification result [24]. In each decision, a Gini Importance of the feature is calculated which determines the best feature to split the data on based on the Gini Index mathematical criteria. RFE functions by recursively eliminating attributes and building the Linear Regression machine learning model on the basis of the selected attributes. It then uses the accuracy of the model that contributes the most to the predictive output of the algorithm. RFE will then rank each feature based on importance with 1 being the most important.

As the second goal of this study was to develop a classification model that would classify stress from data obtained from wearable devices, a ‘modified-dataset’ was created from tailoring original-dataset to present features that were purely relevant to the attributes calculated from the RR intervals recorded from the device. This also aimed to further test the classifiers’ performance on a dataset resembling that generated from the wearable device. Therefore, the relevant features for the modified-dataset were: HR, AVNN, SDNN, RMSSD, pNN50, TP and VLF. The modified-dataset was also the reference dataset for the stress detection application which was used to validate the predictive power of the trained algorithms in a unsupervised system.

### 3.2. Parameter Tuning

In order to achieve the most efficient classification model, hyper-parameter tuning was performed on each algorithm used in this study to determine the best choice of parameters that would yield the highest performance. After generating the baseline for each classifier, where the parameters were set to their default values, a *scikit-learn* library [25] function that loops through a set of predefined hyperparameters and fit the model on the training set was used to perform parameter tuning. Different ranges of each parameter were used in each grid. The outputs from the grid search are the best parameter combinations that give the highest predictive performance which were then compared to their corresponding baseline models. All algorithms in this study were created with the *scikit-learn* library.

#### 3.2.1. K-Nearest Neighbour

K-Nearest Neighbour (KNN) performs classification based on the closest neighbouring training points in a given region [26]; thus, the classification of new test data is dependent on the number of neighbouring labelled examples present at that given location. In order to obtain the best KNN classification model, different values for *k* (number of nearest neighbours) and the *p* value (the power parameter equivalent to the Euclidean distance or Manhattan distance) were investigated. The *k* values investigated ranged from 1 to 30 inclusive, while *p* values could either be 1 (Manhattan distance) or 2 (Euclidean distance). The best parameter values resulted from the grid search are as follows: *k* = 25 and *p* = 1, uniform weights was also selected meaning that all points in each neighbourhood are weighted equally.

#### 3.2.2. Support Vector Machine

The function of the Support Vector Machine (SVM) algorithm is to locate the hyperplane in N-dimensional space (where N represents the number of features) that classifies the data instances into their corresponding class [27] The performance of this algorithm is affected by hyperparameters such as the soft margin regularization parameter (*C*) and kernel, a function that transforms low dimensional inputs space into a higher dimensional space making the data linearly separable.

For the SVM classification model, different *C* values (0.001, 0.01, 0.1, 1, 10, 100 and 1000) and kernels, such as Linear kernel, Polynomial (poly) kernel and Gaussian Radial Basis Function (RBF) kernel were tested. As RBF and poly kernel depends on the gamma (γ, that determines the distance of influence of a single training point) and degree (the degree used to find the hyperplane) parameters respectively, 3 grid searches were carried out for each kernel with γ values of 0.001, 0.01, 0.1, 1, 10, 100 and 1000 and degree values ranged from 1 to 6 inclusive. The best parameter settings resulted to be RBF kernel with γ = 10 and *C* = 100.

#### 3.2.3. Multilayer Perceptron

Multilayer Perceptron (MLP) is a feedforward artificial neural network that was developed to circumvent the drawbacks and limitations imposed by the single-layer perceptron [28]. MLPs are made of at least 3 layers of nodes (input layer, hidden layer and output layer), where each node is connected to every node in the subsequent layer with a certain weight. MLP’s performance, like other machine learning algorithms, is highly dependent on hyperparameter tuning of the following parameters: learning rate coefficient (*h*), momentum (µ) and the size of the hidden layer. *h* determines the size of the weight’s adjustments made at each iteration; *h* values of 0.3, 0.25, 0.2, 0.15, 0.1, 0.1, 0.005, 0.01 and 0.001 were investigated in the grid search. µ controls the speed of training and learning rate; this parameter was set to a range between 0 and 1 with intervals of 0.1. Finally, the size of the hidden layer corresponds to the number of layers and neurones in the hidden layer; the following hidden layer sizes were analysed (10, 30, 10), (4, 6, 3, 2), (20), (4, 6, 3), (10, 20) and (100, 100, 400), where each value represent the number of neurons at its corresponding layer position. A configuration of *h* = 0.001, µ = 0.1 and three hidden layers of 100, 10 and 400 nodes, respectively, proved to be the optimal settings for the model following the grid search.

#### 3.2.4. Random Forest

Random Forest (RF) is an ensemble-based learning algorithm consisting of a combination of randomly generated decision tree classifiers, the results of which are aggregated to obtain a better predictive performance [26]. Based on the parameter tuning grid search performed, the optimal configuration for this algorithm was when the number of trees in the forest (estimators) was set to 300, out of the values 1, 2, 3, 4, 8, 16, 32, 64 and 100 that were tested, with the maximum number of features set to the square root of the total number of features, while the log base 2 of the number of features gave a lower prediction performance.

#### 3.2.5. Gradient Boosting

Gradient Boosting (GB) is also an ensemble-based algorithm composed of multiple decision trees trained to predict new data and where each tree is dependent on one another. This model, which is trained in a gradual, sequential and additive manner, is highly dependent on the learning rate parameter that regulates the shrinkage of the contribution of each tree to the model. The optimal value for this parameter was found to be 0.14 as other learning rate values of 1, 0.5, 0.25, 0.1, 0.05 and 0.01 were also tested in the grid search.

A Naïve Bayes probabilistic algorithm [26] was used as the baseline model for performance comparison between the other more complex algorithms. The configuration for this model was kept as simple as possible by utilising the parameters in their default values as presented by the *GuassianNB* python model.

Furthermore, in order to determine whether there were statistical differences between the investigated models and the baseline model, a One-Way ANOVA statistical test with Tukey’s *post Hoc* comparison was performed on the mean AUROC scores. The null and alternate hypothesis formulated were:

**Hypothesis** **1** **(H1).** *Null Hypothesis: The mean AUROC score for the compared 2 models are equal.*

**Hypothesis** **2** **(H2).** *Alternative Hypothesis: The mean AUROC score for the 2 compared models are not equal, at least AUROC value of one model is different from the other.*

## 4. Results

All results, related to original-dataset and modified-dataset, are described in terms of machine learning metrics such as Area Under Receiver Operator Characteristic Curve (AUROC), Recall/Sensitivity and F1 score [26], including their standard deviation. Every machine learning algorithm was run with a five-fold cross validation. Meanwhile, results from stress classification from data obtained from the Apple Watch are expressed in terms of prediction probability.

### 4.1. Feature Selection on Original-Dataset

**Figure 3:**
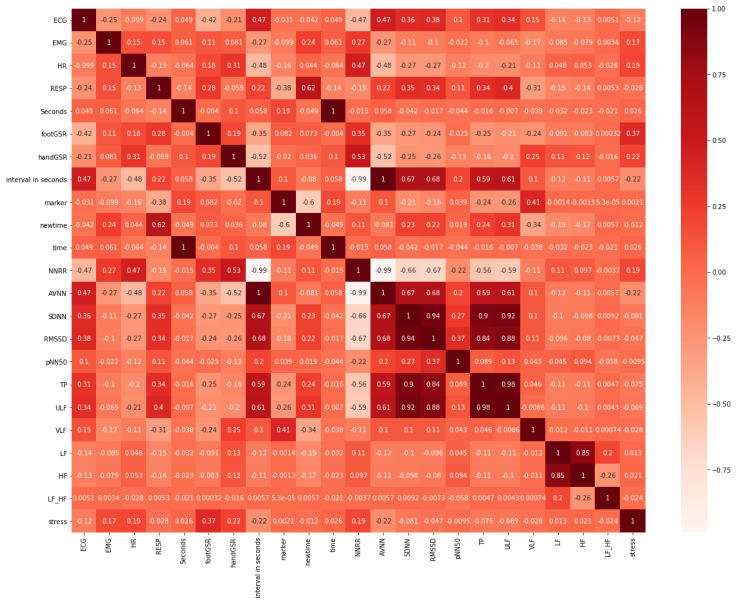
Heat map plot of Pearson’s Correlation Feature Selection performed on original-dataset.

**Figure 4:**
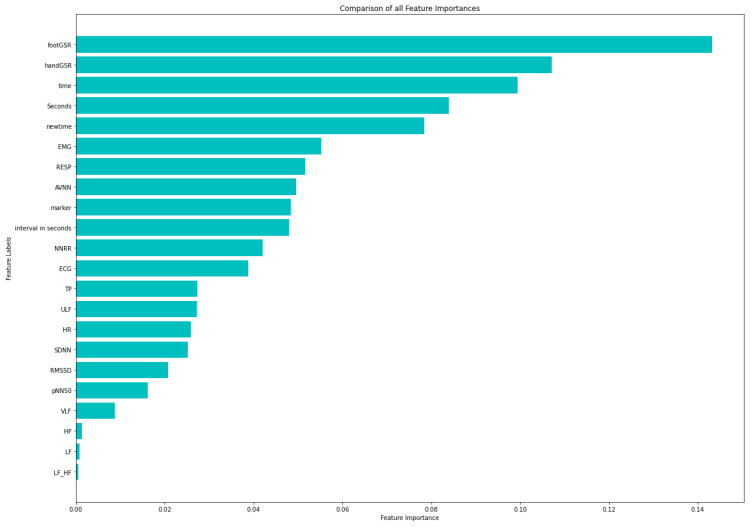
Feature Importance of features from original-dataset using Extra Trees Classifier.

Feature Selection was performed in order to determine the attributes in the dataset that most contribute to the classification task. Figure 3 represents the heat map plot obtained from Pearson’s Correlation. Feature selection scores from RFE, shown in Table 2, indicate that the most relevant features are those with the lowest score. This also shows that the best features (score of 1) were time domain HRV metrics such as RMSSD and AVNN, and frequency domain metrics like TP and ULF, followed by SDNN with a score of 4 (Table 2). Furthermore, Figure 4 illustrates a histogram of the feature importance scores based on the Extra Trees Classifier. Figure 4 shows the Gini Importance of each feature, where the greater the value, the greater the importance of the feature in stress classification.

The common features, from each method, that least contributed to classification or that had the lowest score were dropped from the dataset; these were LF_HF, LF and HF. Additionally, GSR attributes were also dropped because they presented a very strong correlation with stress classification as these were used for stress labelling. Thus, in order to avoid data leakage and overfitting, they were eliminated. Moreover, intuitively redundant features were also dropped like the time related features marker, due to its high number of missing values and EMG, given that it is irrelevant in the context of the smart watch.

### 4.2. Stress Classification on Original-Dataset

In this experiment, stress was classified from bio-signals obtained from subjects who drove under different stress conditions. The results obtained from hyperparameter tuning, illustrated in Table 3, showed that the three best models for the classification task imposed by this dataset were MLP, RF and GB which yielded an AUROC of 83%, 85% and 85% respectively. Thus, the models have more than 83% probability of correctly classifying data instances.

Moreover, MLP and RF presented a Recall of 81% while GB 80% (Table 3); this indicates that at least 80% of the predicted Tue Positive instances are actual positives. Therefore, at least 80% of the instances predicted to be in the stress class have been correctly classified as such. Finally, the F1 scores for MLP, RF and GB are 77%, 78% and 79%, respectively; thus, the model has at least 77% accuracy on the dataset. Figure 3 illustrates the Receiver Operating Characteristics (ROC) curve for all the classifiers investigated in this study.

**Figure 5:**
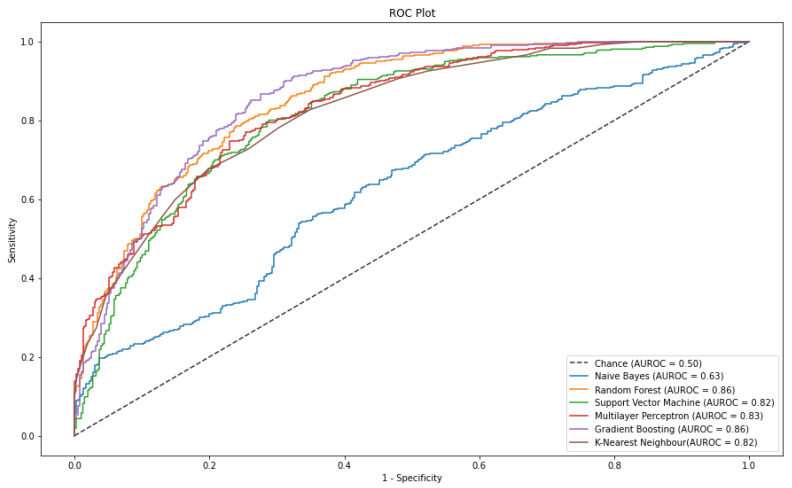
ROC curve plot of each classification model trained on original-dataset. The AUROC scores were achieved by the models during stress prediction of the test dataset from the original-dataset.

Figure 5 consolidates the findings shown in Table 3, illustrating that the models with the greatest ROC area are GB, RF and MLP. It is also visible that this NB model serves as a good baseline model as its ROC curve suggest that its classification is nearly due to chance. A statistical analysis was performed to measure the significance of these results (Table 4).

**Figure 6:**
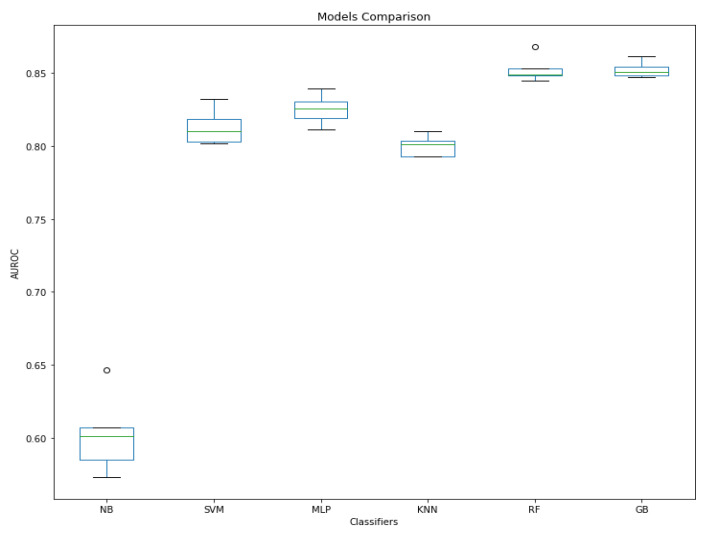
Model performance comparison of machine learning algorithms trained on original-dataset.

Table 4 shows that there was a statistical difference between the AUROC means of all hyperparameter-tuned models and the baseline (NB–AUROC = 60%) as the *p* < 0.05. This confirms that the parameter tuning did improve the model’s performance significantly, and thus, H1 is accepted. Moreover, the Tukey’s comparison test showed that there is a statistically significant difference between the AUROC values of GB and MLP and between MLP and RF (*p* < 0.05). However, the differences between GB and RF are not statistically significant (*p* = 0.9). Figure 6 summarises the results obtained during this experimental series, by illustrating the performance comparison between the hyperparameter-tuned models and the baseline NB model.

### 4.3. Stress Classification on Modified-Dataset

The other objective of this study was to develop a classification model that would classify stress from HRV data obtained from wearable devices. To achieve this, classifiers from Table 3 were used for stress classification of a modified-dataset, which is a modification of the original-dataset but with features that mimic those obtained from the wearable device. Table 5 shows the results obtained during the classification task.

As shown in Table 5, MLP seems to be the overall best performing classifier with 75% AUROC, 80% Recall and 72% F1 score.

**Figure 7:**
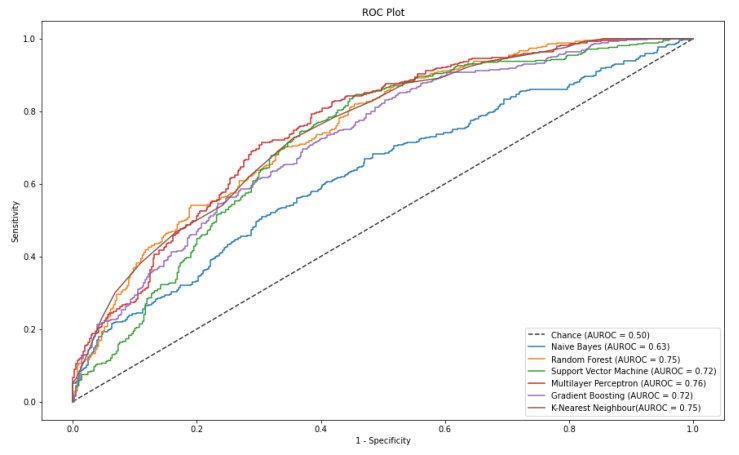
ROC curve plot of each classification model tested on modified-dataset. The AUROC scores were achieved by the models during stress prediction of the test dataset from the modified-dataset.

In addition, Figure 7 illustrates the Receiver Operating Characteristics (ROC) curve for all the classifiers used for the classification of modified-dataset.

Figure 7 shows that the ROC curve from the MLP classifiers seems to be the furthest away from the chance curve and to have the largest area under the curve.

Additionally, a statistical analysis (One-Way ANOVA statistical test with Tukey’s *post Hoc*) of the top three best performing algorithms, obtained from the original-dataset, and their corresponding algorithms, from the modified-dataset, was performed in order to determine their statistical difference. Moreover, these results provided additional insight into which model would be best suited to be implemented in the stress detection application. As shown in Table 6, it is evident that the machine learning algorithms trained on original-dataset are statistically the better performing models (*p* < 0.05), which is expected due to the fact that more information on the dataset is being fed to the model during training.

Furthermore, Table 6 indicates that there is no significant difference in the AUROC values between RF2 and MLP2 (*p* = 0.31). MLP2 was then chosen as the model that will be implemented in the stress detection web application due to its 80% recall score and overall performance. Additionally, another One-Way ANOVA statistical test with Tukey’s *post Hoc* comparison was performed to determine whether there were statistical differences between the models and the Naïve Bayes baseline model. The results determined that there was a statistical difference between the AUROC means of the models and the baseline as the *p* = 0.001 (results not shown).

### 4.4. Stress Classification from HRV Measurements Obtained from Apple Watch

**Figure 8:**
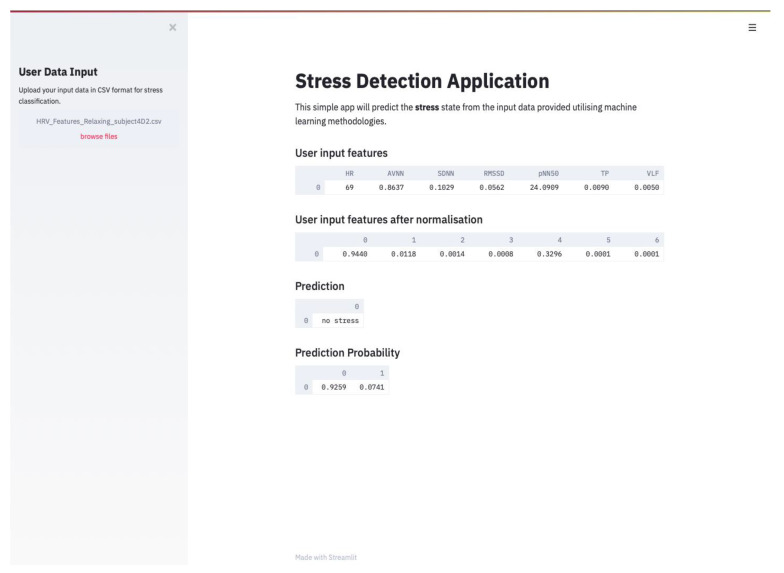
User Interface of the Stress Detection Web Application developed using *Streamlit*.

A simple web application that would perform stress classification on HRV data uploaded by the user (blind-dataset) was developed with the aim to analyse data extracted using wearable devices. The aim of this process was to test the predictive power of the chosen model on data obtained from real participants. The application was developed in Python using the *Streamlit* framework and it is programmed in such way that the user can upload a csv format data, which will be first normalised and then classified as “stress” or “no stress” using the saved MLP model with Recall 80% and AUROC of 75%. Firstly, the application will prompt the user to insert the csv file in the side menu bar. Secondly, the backend code will normalise the input data, so all data instances are within the same range, and display the inserted and normalised data in a tabular format. Thirdly, the normalised data undergoes classification, and the results are displayed as Prediction Probability, shown in Figure 8.

**Figure 9:**
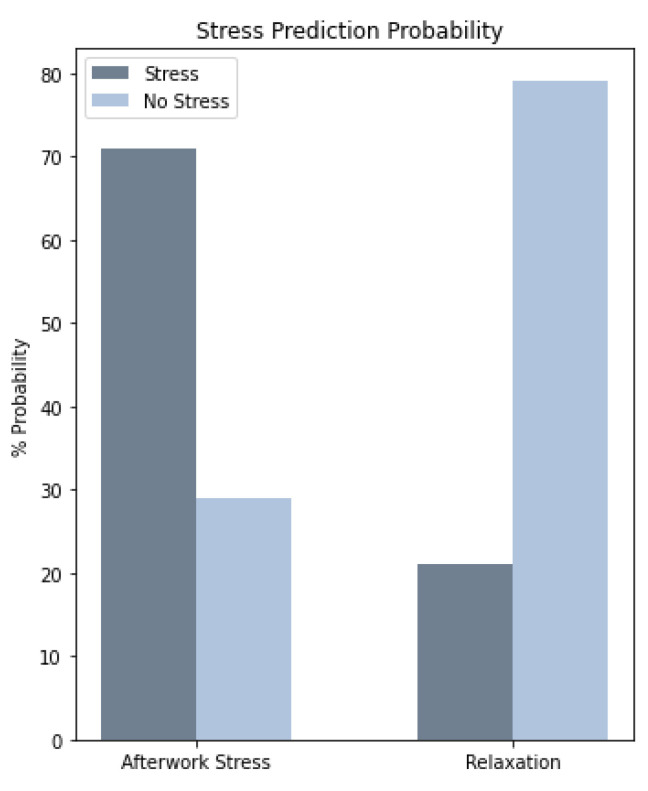
Mean Prediction Probability obtain from the stress detection app with volunteers input data, who were subjected to different stress conditions (after work stress and relaxation).

After running the program with the input data derived from the volunteers, the prediction probabilities for the model to predict an instance as stress or no stress were recorded for the different stress scenarios. Figure 9 summarises the results of this investigation in a bar chart presenting the mean prediction probabilities.

As displayed in Figure 9, the model was able to correctly classify a stress state with a prediction probability of 71 ± 0.1%. Additionally, it was able to achieve a prediction probability of 79 ± 0.3% when the model was presented with a relaxing situation.

## 5. Discussion

Stress has been identified as one of the major causes of automobile crashes [8] and an important player in the development of cardiac arrythmia [7]; therefore, it is important to be able to detect and measure stress in a non-invasive and efficient manner. In this study, to accomplish this, we address the stress detection problem by using traditional machine learning algorithms which were trained on ECG-derived HRV metrics obtained from automobile drivers [18,19].

In this paper, stress classification was performed mainly using HRV-derived features as studies have shown that HRV is impacted during changes in stress levels, given that it is highly controlled by the ANS [10]. Moreover, other investigations proved that RMSSD, AVNN and SDNN were evaluated as being the most reliable HRV metrics in distinguishing between stressful and non-stressful situations [28]. Those findings were also confirmed in this study as shown in Table 2, where AVNN, RMSSD and SDNN were classified as the HRV features with the highest RFE feature importance scores. Therefore, they were considered to be the features that contribute the most in the stress classification performance of the model. This further confirms that HRV features are viable markers for stress detection.

Following hyperparameter tuning, we were able to produce stress classification models with high predictive power. As shown in Table 3, the best 3 models for the classification task imposed by original-dataset were MLP, RF and GB with AUROC of 83%, 85% and 85%, respectively; thus, these classifiers have ~84% probability of successfully distinguishing between the stress and no stress class. In addition, MLP and RF gave Recall scores of 81% while GB of 80%; indicating that ~80% of the predicted positive instances are actual positives. Furthermore, these scores were statistically greater than the Naïve Bayes baseline model (*p* < 0.05) as illustrated in Table 4.

There are very few studies performed on stress classification in drivers using HRV derived features [17,18], although each study took a different approach to the classification problem, the classification yielded similar results. For instance, [17] investigated KNN, SVM-RBF and Linear SVM as their potential classifiers for stress detection. Their results suggested that SVM with RBF kernel was the best performing model by giving an accuracy of 83% [17]. However, more extensive investigation is necessary to corroborate this finding by also considering other classification metrics.

It is also imperative to discuss the fact that stress is a result of a combination of external (environment) and internal factors (e.g., mental health). Thus, stress could be perceived as a subjective mental state; for example, certain situations like a drive in the city or in the highway might not induce the same level of stress in every individual. For instance, individuals suffering from anxiety could feel stressed in such conditions. Additionally, stress could be induced from the invasive apparatus used such as the electrodes placed in different parts of their body and the sensor placed around their diaphragm in [18]; the fact that the subject is aware that they are being monitored for changes in their mental state could also impact their stress levels. For this reason, is important to use less intrusive and everyday devices such as smart watches or mobile phones that are already an essential part of life in this modern society.

In this paper we also aimed to develop a classification model that would detect stress from data obtained from the Apple Watch. For this purpose, the best classifiers trained on original-dataset were tested for the classification of the modified-dataset which presented features that mimic those derived from the wearable device. Table 5 demonstrates that the overall ideal model for the stress classification of HRV features derived from wearable-obtained RR intervals, is MLP with a AUROC of 75% and a Recall of 80%. This was determined based on the Recall score, as in this stress classification task there is a high cost associated with False Negatives. For instance, if an individual’s condition, which is actually stressed, is predicted as not stressed, the cost associated with this False Negative can be high, especially in a medical or driving context which could then lead to a misdiagnosis or a car accident respectively. Therefore, it is imperative to select the model with the highest sensitivity.

Figure 8 shows the user interface (UI) of the simple stress detection web application. The purpose of this was simply to provide a visual UI to demonstrate the software functionality. This could then be implemented into a mobile or car application where the user would be alerted when stress is detected and would prompt them to relax or take breaks.

The blind-dataset, obtained from the volunteers, served as a blind test for the MLP classifier in order to measure its predictive power on unseen data in an unsupervised application system.

When classifying a stressful task, the web application was able to correctly predict stress conditions with a 71% prediction probability. Additionally, it was able to achieve a prediction probability of 79% when the model was presented with a relaxing state. However, it is important to further improve the model’s performance by investigating multiple stress levels in order to obtain more accurate stress detection.

## 6. Conclusions

In this paper, we developed a comparative study to determine the viability of HRV features as physiological markers for stress detection. This was achieved by computing different supervised machine learning models to determine which model can be used to analyse data extracted using wearable devices. The MLP model was considered to be an ideal algorithm for stress classification due to its 80% sensitivity score. The predictive power of this classifier was found to be statistically greater compared to the baseline model created with the Naïve Bayes algorithm with a *p* value of 0.001. This model was then implemented in the unsupervised stress detection application where stress can be detected from blind dataset of HRV features, and extracted from real users using wearable devices under different stress conditions.

A benefit of this study is that there is a need for technologies that would monitor stress in drivers in order to reduce car crashes, as nearly 80% of road incidents are due to drivers being under stress. This project could be the initial steps for tackling this problem. In fact, the algorithm produced in this model could be implemented in smart cars. So, when drivers are experiencing episodes of stress, the automobile could switch to autopilot as well as alert the driver of their state. This implementation could massively reduce traffic accidents as well as reduce the number of fatalities and injuries caused by car crashes.

However, the benefit of this study can also be extended to all applications in which it is important to monitor stress levels e.g., in physical rehabilitation post incident, in temporary or chronic anxiety, in mental health disease, as well as in many ageing conditions. The distribution of smart watches is growing in the population and people appreciate their functionalities. Therefore, wearable devices offer a big opportunity to extract health parameters without an uncomfortable and invasive approach.

We plan that future work should involve the improvement of the classification models by exploring a wider range of parameter values during the hyperparameter tuning process. Additionally, the Deep Learning approach could also be implemented in order to compare its performance in comparison to the supervised models used in this study.

Moreover, another future work we propose is the development of a classifier that would be able to distinguish between different levels stress: high, medium and low. In addition to this, we suggest collecting new real-world ECG data, from which HRV features could be extracted, in order to gain a better insight on the predictive power of the models obtained in this study. This would also provide a more updated dataset compared to that used in this study, dated 2005 [18]. As technologies have advanced, a more accurate ECG recording could be acquired; thus, this would make the classification more accurate and relevant to real world implementations.

Therefore, a natural evolution of this work will require the acquisition of a large dataset through smart watches and in an extensive number of tests involving human subjects e.g., through a driving simulator. Furthermore, it will be important to test the model considering other domains focused to the elderly and health care.

## Figures and Tables

**Table 1:** Time and Frequency Metrics derived from Heart Rate Variability.

Time Domain Metrics	
SDNN	Standard deviation of all NN intervals
SDANN	Standard deviation of the average NN intervals
AVNN	Average of NN intervals
RMSSD	Square root of the mean squared differences of successive RR intervals
pNN50	Percentage differences of successive RR intervals larger than 50 ms
Frequency Domain Metrics	
TP	Total Power—total spectral power of all NN intervals up to 0.004 Hz
LF	Low Frequency—total spectral power of all NN intervals with frequency ranging from 0.04 Hz to 0.15 Hz
HF	High Frequency—total spectral power of all NN intervals with frequency ranging from 0.15 Hz to 0.4 Hz
VLF	Very Low Frequency—total spectral power of all NN intervals with frequencies >0.004 Hz
ULF	Ultra-Low Frequency—total spectral power of all NN intervals with frequencies <0.003 Hz
LF/HF	Ratio of low to high frequency

**Table 2:** RFE feature importance score on original-dataset, the most relevant features have the lowest RFE score.

Feature	RFE Score
EMG	1
HR	1
footGSR	1
handGSR	1
Interval in seconds	1
NNRR	1
AVNN	1
RMSSD	1
TP	1
ULF	1
RESP	2
marker	3
SDNN	4
LF_HF	5
LF	6
ECG	7
pNN50	8
HF	9
newtime	10
time	11
VLF	12
Seconds	13

**Table 3:** Comparison of the predictive performance of the best classifiers obtained from the grid search (trained on original-dataset).

Algorithm	AUROC	Recall	F1 Score
NB	0.60 ± 0.02	0.63 ± 0.04	0.61 ± 0.02
KNN	0.80 ± 0.01	0.76 ± 0.02	0.74 ± 0.01
SVM	0.81 ± 0.01	0.79 ± 0.03	0.77 ± 0.01
MLP	0.83 ± 0.01	0.81 ± 0.07	0.77 ± 0.02
RF	0.85 ± 0.01	0.81 ± 0.03	0.78 ± 0.02
GB	0.85 ± 0.01	0.80 ± 0.02	0.79 ± 0.01

NB, Naïve Bayes; KNN, K Nearest Neighbour; SVM, Support Vector Machine; MLP, Multilayer Perceptron; RF, Random Forest; GB, Gradient Boosting. NB represent the baseline model used as means of comparison for the other complex machine learning algorithm.

**Table 4:** Statistical Evaluation of the machine learning models.

Model A	Model B	mean (A)	mean (B)	diff	se	*p*-Tukey ^1^
GB	KNN	0.852	0.800	0.052	0.009	**0.001**
GB	MLP	0.852	0.825	0.027	0.009	**0.039**
GB	NB	0.852	0.603	0.249	0.009	**0.001**
GB	RF	0.852	0.853	−0.001	0.009	0.9
GB	SVM	0.852	0.813	0.039	0.009	**0.001**
KNN	MLP	0.800	0.825	−0.025	0.009	0.077
KNN	NB	0.800	0.603	0.197	0.009	**0.001**
KNN	RF	0.800	0.853	−0.053	0.009	**0.001**
KNN	SVM	0.800	0.813	−0.013	0.009	0.671
MLP	NB	0.825	0.603	0.222	0.009	**0.001**
MLP	RF	0.825	0.853	−0.028	0.009	**0.036**
MLP	SVM	0.825	0.813	0.012	0.009	0.732
NB	RF	0.603	0.853	−0.250	0.009	**0.001**
NB	SVM	0.603	0.813	−0.210	0.009	**0.001**
RF	SVM	0.853	0.813	0.040	0.009	**0.001**

^1^*p* values in bold represent statistical significance, where *p* < 0.05.

**Table 5:** Predictive performance of machine learning classifiers on modified-dataset.

Algorithm	AUROC	Recall	F1 Score
NB	0.60 ± 0.02	0.69 ± 0.04	0.63 ± 0.02
KNN	0.74 ± 0.02	0.76 ± 0.02	0.71 ± 0.02
SVM	0.74 ± 0.01	0.79 ± 0.02	0.74 ± 0.01
MLP	0.75 ± 0.01	0.80 ± 0.06	0.72 ± 0.02
RF	0.77 ± 0.01	0.74 ± 0.01	0.72 ± 0.01
GB	0.73± 0.01	0.70 ± 0.02	0.70 ± 0.01

**Table 6:** Statistical Evaluation of the machine learning models. Numbers 1 and 2 correspond to original-dataset and modified-dataset respectively.

Model A	Model B	mean (A)	mean (B)	diff	se	*p*-Tukey ^1^
GB 1	GB 2	0.852	0.731	0.121	0.008	**0.001**
GB 1	MLP 1	0.852	0.825	0.027	0.008	**0.011**
GB 1	MLP 2	0.852	0.752	0.100	0.008	**0.001**
GB 1	RF 1	0.852	0.853	−0.001	0.008	0.9
GB 1	RF 2	0.852	0.768	0.084	0.008	**0.001**
GB 2	MLP 1	0.731	0.825	−0.094	0.008	**0.001**
GB 2	MLP 2	0.731	0.752	−0.021	0.008	0.088
GB 2	RF 1	0.731	0.853	−0.122	0.008	**0.001**
GB 2	RF 2	0.731	0.768	−0.037	0.008	**0.001**
MLP 1	MLP 2	0.825	0.752	0.073	0.008	**0.001**
MLP 1	RF 1	0.825	0.853	−0.028	0.008	**0.01**
MLP 1	RF 2	0.825	0.768	0.057	0.008	**0.001**
MLP 2	RF 1	0.752	0.853	−0.101	0.008	**0.001**
MLP 2	RF 2	0.752	0.768	−0.016	0.008	0.313
RF 1	RF 2	0.853	0.768	0.085	0.008	**0.001**

^1^*p* values in bold represent statistical significance, where *p* < 0.05.

## Data Availability

The main dataset used was public “PhysioNet [18]”, as described in the Materials and Methods section; only a few people were involved in the test to validate the feature extraction from apple watch in real condition and the modality of the experiments is reported in this work, therefore the tests can be replicated on further subjects.
